# MicroRNA-208b progressively declines after spinal cord injury in humans and is inversely related to myostatin expression

**DOI:** 10.14814/phy2.12622

**Published:** 2015-11-24

**Authors:** Hanneke Boon, Rasmus J O Sjögren, Julie Massart, Brendan Egan, Emil Kostovski, Per O Iversen, Nils Hjeltnes, Alexander V Chibalin, Ulrika Widegren, Juleen R Zierath

**Affiliations:** 1 Department of Molecular Medicine and Surgery, Section for Integrative Physiology, Karolinska InstitutetStockholm, Sweden; 2 Section for Spinal Cord Injury, Sunnaas Rehabilitation HospitalNesoddtangen, Norway; 3 Department of Nutrition, Institute of Basic Medical Sciences, University of OsloOslo, Norway; 4 Institute of Clinical Medicine, University of OsloOslo, Norway; 5 Department of Hematology, Oslo University HospitalOslo, Norway

**Keywords:** MicroRNA, skeletal muscle, spinal cord injury

## Abstract

The effects of long-term physical inactivity on the expression of microRNAs involved in the regulation of skeletal muscle mass in humans are largely unknown. MicroRNAs are short, noncoding RNAs that fine-tune target expression through mRNA degradation or by inhibiting protein translation. Intronic to the slow, type I, muscle fiber type genes *MYH7* and *MYH7b*, microRNA-208b and microRNA-499-5p are thought to fine-tune the expression of genes important for muscle growth, such as myostatin. Spinal cord injured humans are characterized by both skeletal muscle atrophy and transformation toward fast-twitch, type II fibers. We determined the expression of microRNA-208b, microRNA-499-5p, and myostatin in human skeletal muscle after complete cervical spinal cord injury. We also determined whether these microRNAs altered myostatin expression in rodent skeletal muscle. A progressive decline in skeletal muscle microRNA-208b and microRNA-499-5p expression occurred in humans during the first year after spinal cord injury and with long-standing spinal cord injury. Expression of myostatin was inversely correlated with microRNA-208b and microRNA-499-5p in human skeletal muscle after spinal cord injury. Overexpression of microRNA-208b in intact mouse skeletal muscle decreased myostatin expression, whereas microRNA-499-5p was without effect. In conclusion, we provide evidence for an inverse relationship between expression of microRNA-208b and its previously validated target myostatin in humans with severe skeletal muscle atrophy. Moreover, we provide direct evidence that microRNA-208b overexpression decreases myostatin gene expression in intact rodent muscle. Our results implicate that microRNA-208b modulates myostatin expression and this may play a role in the regulation of skeletal muscle mass following spinal cord injury.

## Introduction

Skeletal muscle has a remarkable capacity to adapt to increased physical activity and exercise training. Ultimately, these changes contribute to increased muscle strength, resistance to fatigue, and more general health benefits of exercise such as reduced risk of metabolic and cardiovascular disease (Egan and Zierath 2013). Physical inactivity opposes the positive effects of acute exercise and long-term training on both the physiological and molecular levels. Spinal cord injury causes extreme physical inactivity, resulting from a varying degree of disruption in nerve connections between the central nervous system and organs below the level of injury. People with spinal cord injury experience severe skeletal muscle atrophy (Hjeltnes et al. 1997). Moreover, skeletal muscle fiber type transformation toward fast-twitch, type II, fibers occurs within the first year after complete cervical spinal cord injury (Kostovski et al. 2013). Such changes reflect a more glycolytic phenotype (Aksnes et al. 1996; Hjeltnes et al. 1997, 1998; Talmadge 2000; Kramer et al. 2006; Long et al. 2011; Verdijk et al. 2012), although the molecular mechanism for these changes in spinal cord injured persons is incompletely described.

MicroRNAs are short, noncoding RNAs that fine-tune target gene expression through mRNA degradation or by inhibiting protein translation, and therefore participate in the regulation of gene expression and adaptive programs (Ambros 2004; Bartel and Chen 2004). Many microRNAs show coordinated expression, either because of close proximity of their genomic location or shared transcriptional regulation. Collectively, a group of related microRNAs fine-tune cellular functions, thus adding an extra layer of complexity to previously known gene regulatory processes (Lanceta et al. 2010). Although first thought to be involved mainly in development (Lee et al. 1993; Wightman et al. 1993), recent insights suggest that microRNAs play a key role in orchestrating stress responses (Leung and Sharp 2010; Mendell and Olson 2012). The genes encoding slow-twitch oxidative type I muscle fiber myosin heavy chains, *MYH7* and *MYH7b*, intronically express microRNA-208b and microRNA-499-5p, respectively (McCarthy et al. 2009). Moreover, mice lacking both microRNA-208b and microRNA-499-p5 show a substantial loss of slow-twitch myofibers (van Rooij et al. 2009). These specific microRNAs are canonical myomirs with important roles in the regulation of skeletal muscle mass (van Rooij et al. 2007; McCarthy et al. 2009). MicroRNA-208b and microRNA-499-5p have nearly identical seed sequences and therefore share many predicted gene targets. One such target is myostatin, a member of the transforming growth factor-*β* (TGF-*β*) superfamily of growth and differentiation factors and an inhibitor of muscle growth (McPherron et al. 1997). Overexpression of myostatin in intact skeletal muscle decreases muscle mass and fiber size (Durieux et al. 2007). Whether microRNA-208b and microRNA-499-5p contribute to skeletal muscle atrophy in spinal cord injury is unknown.

Exercise induces changes in microRNA expression, suggesting a molecular role in coordinating the adaptive response of skeletal muscle to physical activity (Russell et al. 2013). However, the effects of long-term physical inactivity on the expression of microRNAs involved in the regulation of skeletal muscle mass in humans are unknown. Given the known role of microRNA-208b and microRNA-499-5p in determining skeletal muscle size in rodents (van Rooij et al. 2009), we hypothesized that they may be altered, concomitant with skeletal muscle atrophy, in humans after spinal cord injury. Thus, we determined the expression of microRNA-208b, microRNA-499-5p, and myostatin in skeletal muscle obtained from people with complete spinal cord injury. Expression of the different fiber types, microRNAs as well as myostatin was measured after either long-standing or recent (≤1 year) spinal cord injury. Direct modulation of gene target expression was studied in vivo by gene transfer of microRNA-208b or microRNA-499-5p in intact mouse muscle by electroporation.

## Methods

### Ethical approval

The study protocol was explained to the subjects and all participants gave their written informed consent. The Regional Committee for Medical and Health Research Ethics (Health Authority Southern and Eastern Norway) and the Regional Ethical Committee at Karolinska Institutet, Sweden, approved the study protocol. This investigation conforms to the principles outlined in the Declaration of Helsinki.

### Animal care

Animal experiments were approved by the Regional Animal Ethical Committee (Stockholm, Sweden). Mice were maintained on a 12-h light/12-h dark cycle and received standard rodent chow (Lantmännen, Sweden) and access to water ad libitum. Male C57BL/6J mice (12–16 weeks old) were purchased from Charles River (Germany) and acclimatized for at least 1 week before experiments.

### Study participants

The characteristics of the spinal cord injured and control subjects are presented in Table1. Spinal cord injuries are classified by the neurological injury level according to the American Spinal Injury Association Impairment Scale (AIS) (Kirshblum et al. 2011). Two groups of spinal cord injured subjects were recruited for this study: (I) nine males with long-standing (>3 years after spinal cord injury) complete lesion of the cervical spinal cord and (II) seven subjects (six male, one female) with recent, complete cervical spinal cord injury. Participants in group II were studied throughout the first year after spinal cord injury, with biopsies taken approximately 1, 3, and 12 months after injury. Ten healthy males matched for age and body mass index (BMI) were recruited as controls for group I.

**Table 1:** Clinical characteristics of the control and spinal cord injured subjects

	Able-bodied	Spinal cord injured	
	Control	Recent	Long-standing
Male/Female	10/0	6/1	9/0
Age	39 ± 2	33 ± 4	36 ± 3
BMI (kg/m^2^)	22.4 ± 0.4		21.4 ± 1.3
1 month		23.9 ± 1.0	
3 months		24.2 ± 1.1	
12 months		24.6 ± 1.6	
AIS level		A	A

AIS, American spinal injury association impairment scale. Results are mean ± SEM.

### Experimental design of clinical study

All spinal cord injured subjects underwent a clinical examination prior to inclusion. Skeletal muscle biopsies from individuals with a recent spinal cord injury were taken at approximately 1, 3, and 12 months after injury. Biopsies (20–100 mg) were obtained under local anesthesia from the *m. vastus lateralis* and immediately frozen in liquid nitrogen, as previously described (Hjeltnes et al. 1998). Patients had clinically stable vital signs at the time of biopsy.

### Gene transfer by electroporation in intact mouse muscle

Tibialis anterior muscles of adult C57Bl/6J mice were electroporated with either a control plasmid or plasmid encoding for pri-microRNA-208b or pri-microRNA-499-5p (Origene, Rockville, MD) as described previously (Kulkarni et al., 2011). One leg was electroporated with the pri-microRNA expressing plasmid, while the contralateral leg was electroporated with control plasmid. With this design, each individual animal serves as its own control. Seven days after electroporation, both tibialis anterior muscles were removed and stored at −80°C until further analysis.

### RNA isolation and cDNA synthesis

Human skeletal muscle (∼10 mg) was freeze-dried before microscopical removal of fat, blood, and connective tissue. A Trizol method was used for RNA extraction according to the manufacturer’s recommendations (Life Technologies, Stockholm, Sweden). Total RNA concentration was quantified spectrophotometrically at an absorbance of 260 nm (NanoDrop ND-1000 Spectrophotometer; Thermo Fisher Scientific, Waltham, MA). The integrity and purity of the RNA was verified by measuring spectrophotometrically at A260/A280 (>1.8). For quantification of target gene expression, RNA (1 *μ*g) was reverse transcribed to cDNA using the High Capacity cDNA RT kit (Applied Biosystems, Foster City, CA). To quantify microRNA expression, RNA (350 ng) was reverse transcribed using Megaplex Primer Pools (Human Pools) Kit or custom Primer Pool (Applied Biosystems). In both analyses, a reaction without reverse transcriptase was included as a control. The cDNA template was stored at −20°C until subsequent analysis.

### Quantitative real-time PCR for mRNA expression

Relative mRNA expression in human and mouse skeletal muscle was determined by quantitative real-time PCR (StepOnePlus RT-PCR system, Applied Biosystems) using TaqMan primers (Assay IDs MYH7 (Hs01110632_m1), MYH7b (Hs00293096_m1), myostatin (Hs00976237_m1 and Mm01254559_m1), B2M (Hs00984230_m1 and Mm00437762_m1), TBP (Mm00446971_m1), and RPLP0 (4333761F)). All samples were analyzed in duplicate and the relative quantity of different mRNA was calculated after normalization of the data against stable expressed endogenous controls. Optimal endogenous controls for human experiments were chosen based on equal expression between groups as determined by Normfinder software (Andersen et al. 2004). For mRNA expression in human skeletal muscle, the geometrical mean of expression values of B2M and RPLP0 was chosen to normalize gene expression. For mRNA expression in mouse skeletal muscle, the geometrical mean of expression values of TBP and B2M was chosen to normalize gene expression. Fold changes were calculated using the ∆∆Ct method.

### Quantitative real-time PCR for microRNA expression

MicroRNA abundance in human and mouse skeletal muscle was determined by quantitative real-time PCR (StepOnePlus RT-PCR system, Applied Biosystems) using TaqMan primers (Assay IDs hsa-microRNA-208b [PM12444], mmu-microRNA-499-5p [PM1352]). All samples were analyzed in duplicate and the relative quantity of different microRNA transcripts was calculated after normalization of the data against stable expressed endogenous controls. For human skeletal muscle, optimal endogenous controls were chosen based on equal expression between the three study groups as determined by Normfinder software (Andersen et al. 2004). For normalization of microRNA expression in the spinal cord injured subjects, microRNA-133a (PM2246) was chosen. snoRNA202 (PM1232) was chosen as housekeeping gene for normalization of microRNA expression after electroporation in mouse skeletal muscle. Fold changes were calculated using the ∆∆Ct method.

### Statistics

Data are presented as mean ± SEM. Data were evaluated using IBM SPSS Statistics software (IBM Corp., Armonk, NY). A *t*-test for independent samples was performed to identify differences in mRNA/microRNA expression ratio between spinal cord injured (groups I and II) and control subjects. A one-way repeated measure ANOVA with repeated contrast was performed to identify changes in mRNA and microRNA expression levels during the first year after spinal cord injury. When a significant F-ratio was found, a Bonferroni post hoc test for pairwise comparisons was performed to identify differences between time points. Paired *t*-tests were performed to identify changes in mRNA/microRNA expression in rodent muscle after electroporation. Correlation analysis was performed using Pearson’s product moment (*r*) correlation. *P*-values <0.05 were considered statistically significant.

## Results

### Progressive decline in expression of slow-twitch fiber type genes *MYH7* and *MYH7b* after spinal cord injury

**Figure 1:**
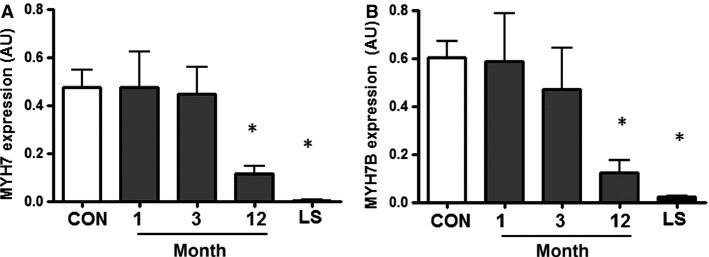
*MYH7* (A) and *MYH7b* (B) gene expression in able-bodied control subjects (CON – white bar), and people with complete cervical spinal cord injury studied 1, 3, or 12 months post injury (gray bars) or after long-standing injury (LS – black bar). *n *=* *7–10, *P <* *0.05 for spinal cord injury versus able-bodied controls.

Relative mRNA expression of slow-twitch fiber type genes *MYH7* and *MYH7b* progressively declined with time after injury (Fig.1). Expression of *MYH7* was decreased ∼76% at month 12 after spinal cord injury compared to the able-bodied control group (*P* < 0.001, Fig.1A). In individuals with long-standing injury, *MYH7* expression was decreased to <1% of that observed in the able-bodied control group. *MYH7b* expression was decreased 80% at month 12 after injury, whereas in individuals with long-standing injury, expression level was 3% of that observed in the able-bodied control group (*P* < 0.001, Fig.1B).

### Expression of microRNA-208b and microRNA-499-5p is decreased after spinal cord injury and correlates with expression of host genes *MYH7* and *MYH7b*

**Figure 2:**
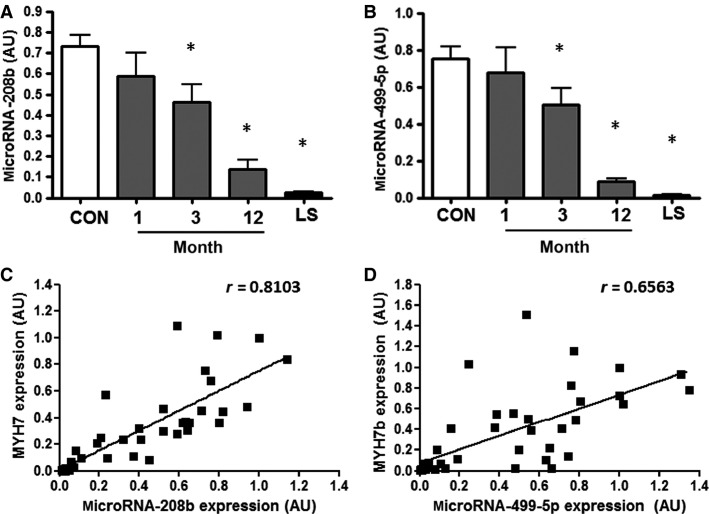
MicroRNA-208b (A) and microRNA-499-5p (B) expression in able-bodied control subjects (CON – white bar), and people with complete cervical spinal cord injury studied 1, 3, or 12 months post injury (gray bars) or after long-standing injury (LS – black bar). *n *=* *7–10, *P <* *0.05 for spinal cord injury versus able-bodied controls. Correlation between (C) *MYH7* gene expression and microRNA-208b expression and (D) *MYH7b* gene expression and microRNA-499-5p expression in people with spinal cord injury and able-bodied control subjects.

Expression of microRNA-208b and microRNA-499-5p progressively declined with time after spinal cord injury (Fig.2). MicroRNA-208b expression was decreased ∼35% and ∼80% at months 3 and 12 after injury, respectively, compared to the able-bodied control group (*P* < 0.02 and *P* < 0.001, Fig.2A). In individuals with long-standing injury, microRNA-208b expression declined to 3% of that observed in the able-bodied control group (*P* < 0.001), with expression levels being close to the limit of detection. MicroRNA-499-5p expression was approximately 33 and 90% lower at months 3 and 12 after injury, respectively, compared to the able-bodied control group (*P* < 0.05 and <0.001, Fig.2B). In individuals with long-standing injury, level of microRNA-499-5p was 2% of that observed in the able-bodied control group (*P* < 0.001). Furthermore, expression of both microRNA-208b and microRNA-499-5p correlated with expression of their respective host genes, namely *MYH7* and *MYH7b* (*r *=* *0.810 and 0.656, respectively, *P *<* *0.001 Fig.2C-D).

### Myostatin gene expression inversely correlates with microRNA expression

**Figure 3:**
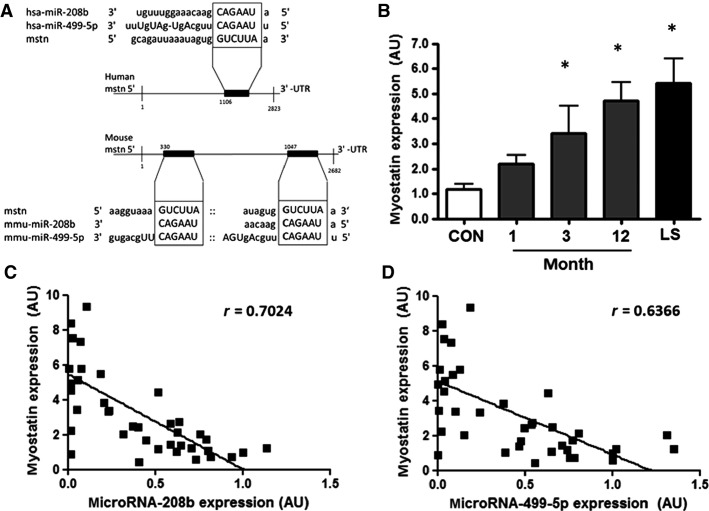
(A) Predicted microRNA seed/mRNA 3′-UTR interaction between microRNA-208b and microRNA-499-5p within the 3′-UTR of the human and mouse myostatin transcripts according to microRNA.org. (B) Myostatin mRNA expression in able-bodied control subjects (CON – white bars), and people with complete cervical spinal cord injury studied at 1, 3, or 12 months post injury (gray bars) or after long-standing injury (LS – black bars). *n *=* *7–10, *P <* *0.05 for spinal cord injury versus able-bodied controls. Correlation between (C) myostatin gene expression and microRNA-208b expression and (D) myostatin gene expression and microRNA-499-5p expression in people with spinal cord injury and able-bodied controls.

The myostatin 3′ untranslated region (UTR) includes a mouse to human conserved seed-binding site for microRNA-208b and microRNA-499-5p (Fig.3A). In the mouse myostatin gene 3′-UTR there is an additional binding site for microRNA-499-5p (Fig.3A). Expression of myostatin increased three- to fourfold at months 3 and 12 after spinal cord injury, respectively, compared to the able-bodied control group (Fig.3B). In individuals with long-standing injury, myostatin mRNA expression increased fivefold compared with the able-bodied control group. Myostatin expression was inversely correlated with microRNA-208b and microRNA-499-5p expression in all groups (*r *=* *0.702 and 0.637, respectively, *P* < 0.001, Fig.3C–D).

### Overexpression of microRNA-208b in mouse skeletal muscle decreases myostatin gene expression

**Figure 4:**
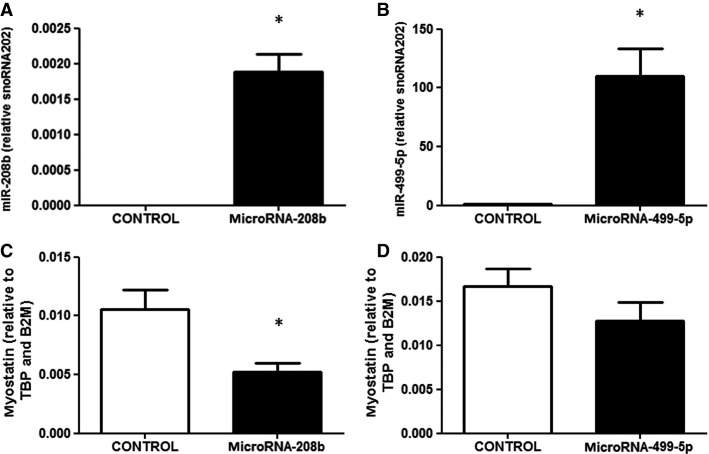
(A) Expression of microRNA-208b in mouse skeletal muscle after electroporation with either microRNA-208b or a control plasmid. (B) Expression of microRNA-499-5p in mouse skeletal muscle after electroporation with either microRNA-499-5p or a control plasmid. (C) Myostatin mRNA 1 week after microRNA-208b overexpression. (D) Myostatin mRNA 1 week after microRNA-499-5p overexpression. *n *=* *6–7 mice, *P <* *0.05 for skeletal muscle electroporated with microRNA plasmid (black bars) versus control plasmid (white bars).

Intact tibialis anterior mouse muscle was electroporated with either a control plasmid or plasmid encoding for pri-microRNA-208b or pri-microRNA-499-5p. Thus, the contralateral leg served as a control. One week after electroporation, skeletal muscle was harvested and expression of the respective microRNA was measured. With this method, we achieved an overexpression of microRNA-208b or microRNA-499-5p in predominantly glycolytic, type II, tibialis anterior muscle, respective to control muscle (Fig.4A–B) that was comparable to levels measured in nontransfected oxidative soleus muscle (data not shown). Relative mRNA expression of myostatin decreased in skeletal muscle overexpressing microRNA-208b, but not microRNA-499-5p (Fig.4C–D).

## Discussion

Here, we report that skeletal muscle expression of microRNA-208b and microRNA-499-5p, as well as their host genes *MYH7* and *MYH7b*, decline progressively during the first year after cervical spinal cord injury in humans, with changes maintained in long-standing injury. We also found that microRNA levels were inversely correlated with an increase in the expression of myostatin, an inhibitor of muscle growth. Finally, overexpression of microRNA-208b, but not microRNA-499-5p, in adult rodent skeletal muscle decreased myostatin gene expression.

Spinal cord injury leads to profound skeletal muscle atrophy and a slow-to-fast muscle fiber type transition (Aksnes et al. 1996; Hjeltnes et al. 1997; Spungen et al. 2003; Long et al. 2011; Kostovski et al. 2013). These morphological changes arise from a combination of both muscle disuse and decreased nerve activity (Bodine 2013). With spinal cord injury, not only is skeletal muscle strength and resistance to fatigue affected, but also the metabolic characteristics including glycogen storage capacity and whole-body glucose tolerance are impaired (Duckworth et al. 1980; Bauman and Spungen 1994; Wahman et al. 2010). Given that skeletal muscle plays vital roles in locomotion, heat production during periods of cold stress, and overall metabolism (Zierath and Hawley 2004), understanding the molecular mechanisms underpinning the plasticity of this tissue to a variety of physiological and pathophysiological stressors may uncover new ways to preserve muscle mass to prevent functional and metabolic impairments.

MicroRNAs are short, noncoding RNAs that inhibit mRNA translation or induce mRNA degradation by binding to complementary, or near-complementary, sequences in the 3′-UTR of mRNA targets (Bartel 2004). The so-called myomir family is a group of microRNAs that includes microRNA-208a, microRNA-208b, and microRNA-499-5p, which fine-tune muscle morphology and function (McCarthy 2011). In particular, microRNA-208b and microRNA-499-5p play a role in the regulation of skeletal muscle fiber type and skeletal muscle mass (McCarthy et al. 2009; van Rooij et al. 2009). Together with microRNA-208a, these microRNAs are encoded by introns of myosin genes (*MYH6*, *MYH7*, *MYH7b*). MicroRNA-208b and microRNA-499-5p have similar seed regions that overlap by six bases, indicating that they share several target genes (van Rooij et al. 2009). Although these microRNAs have been implicated in the control of skeletal muscle fiber type (van Rooij et al. 2009; Gan et al. 2013), they may also be important for the regulation of muscle mass (van Rooij et al. 2007; McCarthy et al. 2009). Hindlimb suspension for 28 days leads to skeletal muscle atrophy, concomitant with decreased expression of microRNA-208b and microRNA-499-5p in rat soleus muscle (McCarthy et al. 2009). Here, we provide evidence that changes in the expression of these microRNAs are coincident with skeletal muscle atrophy in humans after complete spinal cord injury (Hjeltnes et al. 1997).

Expression of microRNA-208b and microRNA-499-5p, as well as the slow myosin genes to which these particular microRNAs are intronic, decline progressively within the first year after spinal cord injury. Myostatin, a target gene of microRNA-208b and microRNA-499-5p and an inhibitor of muscle growth, is upregulated and inversely correlated with the expression of these microRNAs. Our findings corroborate earlier studies describing a feedback loop regulating skeletal muscle mass (McCarthy et al. 2009; van Rooij et al. 2009), whereby decreased expression of *MYH7* and *MYH7b* attenuate the expression of the encoded myomirs, microRNA-208b, and microRNA-499-5p. This in turn upregulates their target genes, which includes myostatin, a transcriptional repressor of muscle growth (McPherron et al. 1997; Lee 2004; Schuelke et al. 2004; Durieux et al. 2007). Nevertheless, the regulation of intronic microRNA expression is likely to be complex, as about one-third of all intronic microRNA regions are predicted to have their own promotor and are expressed independent of their respective host gene (Monteys et al. 2010).

Generally, several microRNAs work in coordination to regulate certain functions by targeting more than one gene. MicroRNA-27a/b regulates myostatin expression and induces skeletal muscle atrophy in rodents (McFarlane et al. 2014). Conversely, microRNA-23a overexpression protects adult muscle tissue from atrophy (Wada et al. 2011). Muscle mass is a function of both protein synthesis and protein breakdown processes. Thus, microRNAs, together with more traditional regulatory mechanisms affect overall protein balance to modulate skeletal muscle mass. Indeed, microRNA-1 and -133 (Elia et al. 2009; Hua et al. 2012), microRNA-125 (Ge et al. 2011), microRNA-206 (Yan et al. 2013), and microRNA-486 (Small et al. 2010) modulate IGF-1/PI3K/AKT signaling, as well as protein synthesis (Wang 2013). Nevertheless, the role of microRNA-208b and microRNA-499-5p in the control of protein synthesis in human skeletal muscle has yet to be determined.

The expression and regulation of myostatin in humans following spinal cord injury is underexplored. In rodent skeletal muscle, myostatin expression increased during the first 14 days after nerve crush injury, at a time when muscle cross-sectional area and wet weight mass also declined (Liu et al. 2007). With more chronic or severe skeletal muscle wasting arising from chronic obstructive pulmonary disease (COPD), HIV-induced cachexia or 25 days of bed rest, serum levels of myostatin are increased (Gonzalez-Cadavid et al. 1998; Zachwieja et al. 1999; Ju and Chen 2012). Conversely, 2 weeks of leg immobilization in humans is associated with decreased myostatin protein abundance (Snijders et al. 2014). The decrease in myostatin expression in response to an acute stimuli for muscle wasting, such as leg immobilization (Snijders et al. 2014), may represent a compensatory mechanism to attenuate the loss of muscle mass. With more severe chronic physical inactivity or reduced innervation, as seen with spinal cord injury, such a protective mechanism might be lost and consequently myostatin levels may be upregulated (Gonzalez-Cadavid et al. 1998; Zachwieja et al. 1999; Ju and Chen 2012). A cross-sectional study of human skeletal muscle for 3 months to 30 years after spinal cord injury provides evidence that myostatin mRNA, but not protein abundance, is reduced compared to able-bodied healthy people (Leger et al. 2009). Thus, our finding of increased myostatin gene expression in both recent and long-standing spinal cord injury is discordant with this previous study. The divergence may be explained by the differences in the severity and level of injury, or degree and length of inactivity. For example, in nerve crush injury (Liu et al. 2007) or complete spinal cord injury, the signaling network between the brain and skeletal muscle is entirely lost. This differs from immobilization where brain–skeletal muscle cross talk remains intact (Snijders et al. 2014). Although myostatin has been validated as a target of both microRNA-208b (Callis et al. 2009) and microRNA-499-5p by luciferase assay (Bell et al. 2010), the in vivo regulation is unknown. Here, we demonstrate that overexpression of microRNA-208b, though not microRNA-499-5p, in rodent skeletal muscle decreased myostatin gene expression in vivo. Thus, the feedback loop is complex and alterations in microRNA-208b can also directly affect myostatin gene expression.

The loss of skeletal muscle mass and function due to spinal cord injury can be mitigated by exercise training such as neuromuscular electrical stimulation (Hjeltnes et al. 1998; Shields and Dudley-Javoroski 2006). These training regimes increase the abundance of key proteins involved in glucose metabolism (Hjeltnes et al. 1998), and mediate long-lasting improvements in skeletal muscle mass, fatigue index, and bone mineral density (Shields and Dudley-Javoroski 2006). Moreover, electrical stimulation increases expression of sarcomeric proteins that mediate the slow-twitch phenotype such as *MYH7*, while expression of atrophic genes like myostatin is reduced (Adams et al. 2011; Petrie et al. 2014). While acute and chronic electrical stimulation training consistently increases MYH7, expression of its intronic microRNA-208b have not been assessed. Collectively these studies reveal that skeletal muscle retains a high degree of plasticity. Moreover, they highlight the importance of myostatin and *MYH7* as key regulators of skeletal muscle function in spinal cord injury.

In conclusion, skeletal muscle expression of microRNA-208b and microRNA-499-5p progressively declined within the first year after cervical spinal cord injury in humans, with changes maintained in long-standing injury. Moreover, myostatin expression was inversely correlated with microRNA-208b and microRNA-499-5p in human skeletal muscle following spinal cord injury, coincident with skeletal muscle atrophy. We also report that in vivo overexpression of microRNA-208b, but not microRNA-499-5p directly reduced myostatin gene expression in mouse skeletal muscle. Collectively, our results provide evidence to suggest that microRNA-208b plays a role in inducing myostatin expression after spinal cord injury in humans and may contribute to the complex regulation of skeletal muscle atrophy in tetraplegia.
